# A maturity model framework for federated networks of trusted research environments

**DOI:** 10.3389/fdgth.2026.1699125

**Published:** 2026-05-15

**Authors:** Jasper H. C. Luong, Jillian Beggs, Jason Ferris, Koh Furuta, Alain-Dominique Gorse, Emily Jefferson, Khin Mi Mi Aung, Clair Sullivan, Xing Yi Woo, The HEALTHWISE Consortium, Io Hong Cheong, Zisis Kozlakidis, Philip R. Quinlan, Tim Beck

**Affiliations:** 1 Department of Microbiology and Immunology, University of British Columbia, Vancouver, BC, Canada; 2 University of Dundee, Dundee, United Kingdom; 3 Queensland Digital Health Centre, The University of Queensland, QLD, Brisbane, Australia; 4 Chiba Medical Center, Clinical Laboratory, Chiba, Japan; 5 Data Science Collaborative Research Platform, The University of Queensland, QLD, Brisbane, Australia; 6 Institute for Infocomm Research (I²R), Agency for Science, Technology and Research (A*STAR), Singapore, Singapore; 7 Digital Intelligence Medical Research Institute, Hainan International Medical Center, Shanghai Jiao Tong University School of Medicine, Qionghai City, Lecheng Medical Tourism Pilot Zone, Hainan Province, China; 8 International Agency for Research on Cancer, World Health Organization, Lyon, France; 9 Centre for Health Informatics, NIHR Nottingham Biomedical Research Centre, University of Nottingham, Nottingham, United Kingdom

**Keywords:** electronic health records, federated networks, health data, maturity model, trusted research environments

## Abstract

**Introduction:**

A Trusted Research Environment (TRE) is a highly secure computer system where sensitive data is stored that researchers can access remotely and make use of in a safe setting. TREs can form federated networks when they have overlapping objectives, such as providing data access to the same researchers for the same project. However, there is variation in the design and implementation of TREs. This makes it difficult for these systems to interoperate without guidance on the technical pathway that should be followed to achieve federation maturity, and resources to assess the current state of maturity.

**Methods:**

We evaluated international maturity models for measuring the capabilities of life sciences and healthcare federated digital infrastructures to identify essential components for federation. Merging synonymous and related components resulted in a maturity model framework consisting of six discreet domains that are required for optimal TRE federation.

**Results:**

The framework comprises of one governance structure domain (governance and policies), one service delivery domain (operations and performance), three technical domains (data management and security, operational infrastructure, clinical/research infrastructure) and one engagement strategy domain (outreach and communication). The TRE community in the UK, convened by DARE UK, have developed a technical blueprint for deploying standalone TREs (called SATRE) and a technical Federated Architecture Blueprint (FAB) for enabling federation between TREs. These blueprints align with many of the maturity model framework's domains.

**Discussion:**

We suggest criteria for implementing the maturity model framework domains and for assessing their relative levels of maturity, and where appropriate reference the recommendations from SATRE and FAB. The maturity model framework can be used as a foundation for assessing the governance, operational and technical readiness of TREs when joining federated networks.

## Introduction

**Figure 1:**
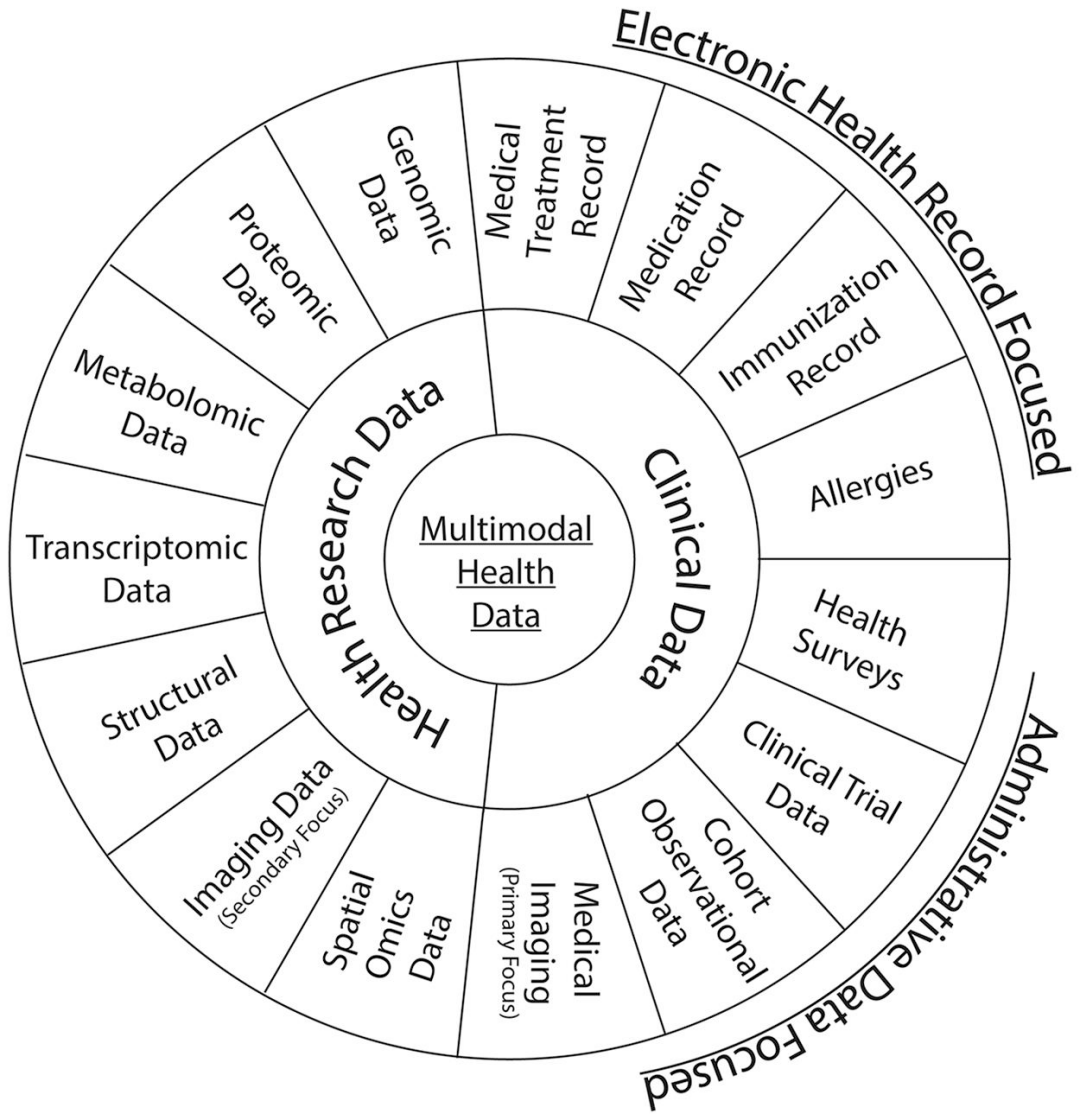
Multimodal health data types in health research and clinical settings. A visual overview of the diverse data types contributing to a comprehensive “Multimodal Health Data” ecosystem. The concentric circles group related categories, including but not limited to -omics data, structural data, imaging data, EHR-focused data (patient centric), and admin-focused data (systems centric) (1, 11–16).

The ever-changing landscape of clinical and health research generates new knowledge while producing large volumes of data that increasingly require sophisticated tools and appropriate environments for data management, analysis, and interpretation. In healthcare, the data are multimodal and include, but are not limited to, the types outlined in Figure 1. Multimodal health data holds great potential collectively in advancing the understanding of diseases, developing treatments and enabling personalized medicine (1). Moreover, population-based and healthcare cohort data are used to identify incidence and trends of different diseases, primarily for non-communicable diseases, and provide opportunities to identify emergence of rare diseases or elevate drug safety (2, 3). Figure 1 outlines the diversity of health data and emphasizes the importance of integrating these varied sources to enable a holistic foundation for research. This also includes primary vs. secondary use data, such as imaging data that was originally used for diagnostics and administrative purposes by a healthcare provider, compared to secondary reuse of that imaging data for research purposes. However, such data requires a mature infrastructure and interoperability among various digital healthcare initiatives and Electronic Health Records (EHRs) (4–8). When managed effectively, the extended applications of these data include predictive modeling and machine learning, as well as generative artificial intelligence (AI). Connecting data sources from geo-diverse systems can enhance research capacity and potential (2, 9, 10).

**Figure 2:**
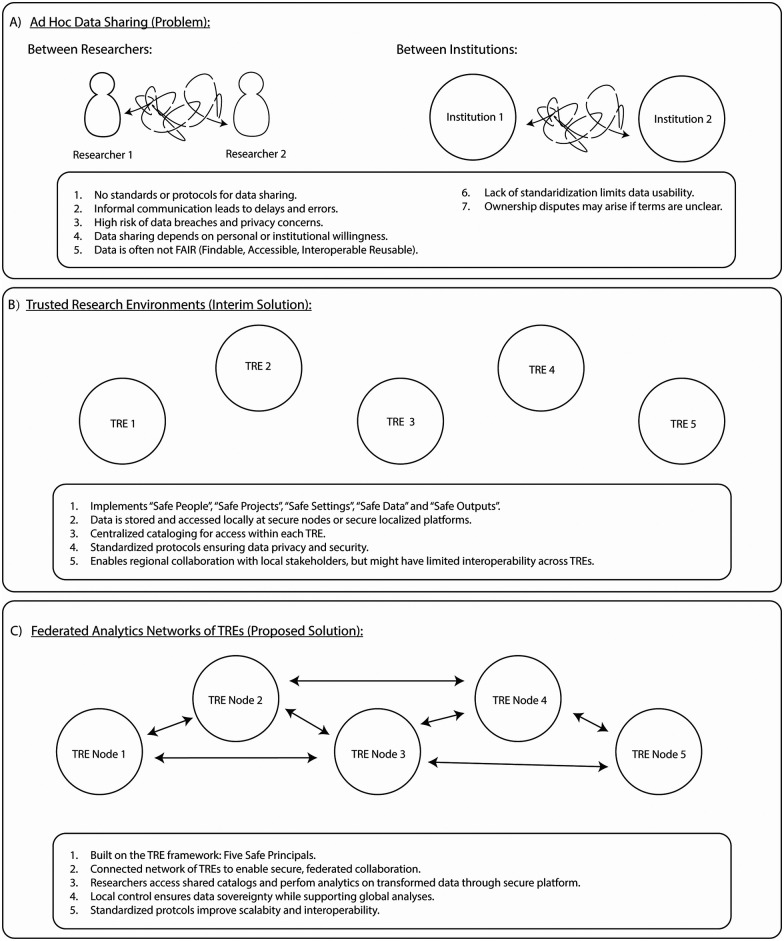
Evolution of data sharing solutions, from *ad hoc* methods to federated TRE networks. The figure illustrates the progression from unstructured, informal data sharing (*ad hoc* data sharing) between researchers and institutions **(A)**, characterized by inefficiencies, security risks, and a lack of standardized protocols, to the use of TREs as an interim solution **(B)** (34–39). TREs offer secure, localized data storage and analysis based on the “Five Safes” principles but are limited by their isolated nature (40). The final stage, Federated Networks of TREs **(C)**, connects multiple TREs into a federated system, enabling secure, collaborative analytics across nodes. This network preserves local data control while supporting interoperability, scalability, and global research efforts through standardized protocols.

The increasing complexity and sensitivity of data has introduced significant challenges to its storage, processing, and utilization for research. These challenges include ensuring secure storage, addressing privacy and ethical concerns, and achieving interoperability across different formats and tools (17–19). The sharing and/or access of such data for different research initiatives would also raise concerns without data sharing agreements and mature security practices, as ownership of every single byte could be disputed as personal property of either the source, or the collector, raising ethical, legal, and social issues (ELSI) (20, 21). The potential problems of sharing these types of health data between researchers and institutions are illustrated in Figure 2A. A complex array of factors need to be considered on an *ad hoc* basis to ensure compliance with data security, privacy, ethical and legal requirements.

To address these issues, Trusted Research Environments (TREs) have emerged as a solution for storing, accessing and analyzing sensitive data provided by different organizations (Figure 2B). The foundational basis of TREs is the “Five Safes” framework: safe people, safe projects, safe settings, safe data and safe outputs. This model was first developed by the Office of National Statistics (22), with Principles and Best Practice Guidance developed by Health Data Research (HDR) UK (23). TREs can provide approved researchers with secure access to sensitive data using state-of-the-art authentication and authorization access protocols and safety measures (24). TREs provide access to the minimum amount of data required for a project approved by a Data Access Committee. Examples of TREs can be found on HDR UK's Health Data Research Gateway portal, a platform that allows access to data collections from across the UK (25, 26).

For TREs to become “business as usual”, several issues remain to be resolved. The demand-driven development of TREs has resulted in several potential drawbacks of the current system for users, such as a lack of standardization of what is required for a TRE, immature infrastructure to find and browse relevant data, varying geo-specific data security concerns and geopolitical or geo-ethical constraints on data acquisition and safe access across regional or national borders. The above drawbacks could, however, be potentially resolved through the adoption of federated networks for secure data access and analysis (Figure 2C). There is potential for individual TREs to interoperate to form federated networks with shared technical components, such as a common authentication and authorization infrastructure, and agreed data quality and preparation processes, such as using a common data model for structuring the data (4). Federated analytics and federated learning initiatives support leading edge research across jurisdictions, and their ability to gain access to multiple sources of data can mitigate any sampling biases in research outcomes (27).

**Figure 3:**
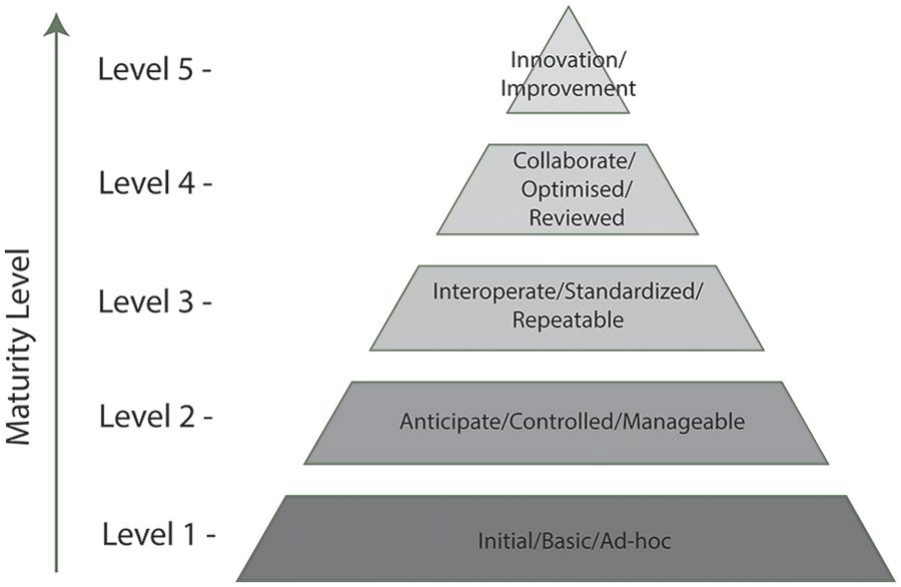
Common key-terms used for maturity models in healthcare. Keywords based on Gomes and Romão, 2018’s discussion on information system maturity models in healthcare, from least to most mature (Level 1—Level 5) (28).

Despite the potential of federated TREs, there is no standardizing framework to evaluate the maturity of such a federated network. Within healthcare, there are maturity models that evaluate information systems (Figure 3), while some federated networks in the field of life sciences digital infrastructure have created custom maturity models. These include: i) ELIXIR, which is the European research infrastructure for life sciences data, and provides a maturity model for human data infrastructure; ii) Federated European Genome–Phenome Archive (EGA), which is a network of national nodes, and provides a maturity model for genomic data infrastructure; iii) Beyond One Million Genomes (B1MG) project, which is a network of genetic and clinical data providers across Europe, and provides a maturity model for healthcare systems (28–31). While these maturity models are applicable to federated infrastructure, they are not designed for use with TREs. The UK Standard Architecture for Trusted Research Environments (SATRE) project (32) provides an architecture specification for standalone TREs, however this in isolation does not include requirements for federation. The DARE UK Federated Architecture Blueprint (FAB) sets outs high-level requirements for establishing secure networks of UK TREs and defines methods for securely moving data between TREs and sending analytics from one TRE to another. Many of the FAB requirements are mapped to one or more of the SATRE specification descriptors (33).

There is a lack of maturity models that are generically applicable to TREs, and no maturity models exist that may be applicable to a federated TRE network. The aims of this manuscript are to: i) compare the maturity level models of existing federated networks for evaluative criteria and domains, and ii) to produce an evaluative maturity model framework for federated networks consisting of TREs.

## Methods

A comparative analysis was performed of the maturity level models for ELIXIR, Federated EGA and B1MG (29–31, 41). These three federated networks are primary initiatives within Europe with dedicated strategies and infrastructure to support data access and analysis of sensitive human data across borders. Moreover, there has been collective alignment in terms of interoperability amongst these networks, making them an appropriate foundation to use as a reference. ELIXIR, Federated EGA and B1MG have maturity models to evaluate human data infrastructure, genomic data infrastructure, and healthcare systems respectively. Although not designed for assessing TREs, these maturity models are applied to digital infrastructure with some similar characteristics to TREs, for example they need to manage sensitive human data. Each network's maturity model consists of domains, subdomains, indicators and precisely defined maturity assessment levels. The domains from each network's maturity model were characterized into specific categories, which were in turn grouped into the initial domains listed in Figure 2. The indicators from each network's maturity model domains and subdomains were aligned against similar indicators from the other networks by their key terms, objectives, and assessment levels, and grouped based on their similarities. Common overlaps were then identified based on the assessment level descriptions and an initial consolidated set of domains relevant to TREs extracted. The common key phrases from each maturity model are shown in Supplementary Table S1 where they are organized into the five common healthcare maturity levels used in maturity level models (Figure 3).

After the initial maturity model evaluation, we compared the grouped domains and subdomains with the proposed HDR UK Capability Maturity Model for TREs that was published in response to the Goldacre Review (40). It presents four levels of maturity for TREs implementing the “Five Safes” principles to support federated analytics. Domains were assessed for complementarity to the Capability Maturity Model and new domains that are unique to TREs were identified. Finally, the SATRE TRE architecture spreadsheet checklist (42) and the DARE UK FAB requirements list were mapped to the domains to provide suggested criteria for implementing each of the mapped domains.

## Results

### Existing federated network maturity models

ELIXIR coordinates bioinformatic resources across its network of national nodes (institutions that facilitate data exchange or communication) and a centralized hub to provide life scientists access to data, tools and training, with a focus to promote standardization and collaboration between organizations (43). FEGA is part of the ELIXIR infrastructure and is a network initiative that focuses on providing secure access to human genomic and phenotypic data to support research worldwide (44). Lastly, B1MG was founded to improve disease prevention and promote the benefits of personalized medicine across the healthcare system in Europe (45). All three examples of federated networks have a joint approach to collectively improve the research, standards and outcomes of its members through means of collaboration, providing opportunities for its members to find and reuse previously inaccessible data from their network peers.

### Comparison of federated networks

Each federated network organizes its maturity model to best suit its objectives and mission, with varying domains and subdomains. Indicatively, ELIXIR's Human Data Infrastructure Maturation Model consists of seven domains and 27 subdomains in total, related to both institutional and core functionality indicators. The domains listed are ELSI; organization; data discoverability; data reception; storage and interfaces; data management access and processing. These domains are included alongside the domains of the other two federated networks in Table 1.

**Table 1:** The comparative analysis of the federated network maturity models, the domains and their descriptors for each maturity level.

Federated Network	ELIXIR	FEGA	B1MG
Domains	ELSI	Governance, Strategy and Sustainability	Governance & Strategy
	Organization	Legal	Investment & Economic Model
	Data Discoverability	Data & Metadata Management	Ethics, Legislation & Policy
	Data Reception	Technical Infrastructure	Public Awareness & Acceptance
	Storage & Interfaces	Operations Support	Workforce skills & Organization
	Data Management Access	Communications, Community Building and Engagement	Clinical Organization, Infrastructure & Tools
	Processing		Clinical Genomics Guidelines & Infrastructure
			Data Management, Standards & Infrastructure
Number of Maturity Levels	4 (1–4)	5 (1–5)	5 (1–5)
Total Number of Indicators under all subdomains	28	36	49

The comparative analysis of the federated network maturity models, the domains and their descriptors for each maturity level, resulted in twelve general evaluation categories as described in Table 2. These include Governance (organization/ELSI); Collaborative Initiatives; Economics; Performance; Data Protection/Privacy; Data Management & Security; Physical Infrastructure; Technical Infrastructure; Outreach and Communications; Operational Environment; Clinical/Research Infrastructure; Novelty Uptake & Review. These categories were not consistent across the federated networks' maturity models but are overlapping according to their descriptors. Subsequent descriptors from the HDR UK Capability Maturity Model were fitted into all the categories to identify the gap descriptors needed to generate a maturity model for a federated TREs network.

**Table 2:** Comparative analysis for the ELIXIR, FEGA and B1MG maturity models sorted into twelve general domains: governance; collaborative initiatives; economics; performance; data protection/privacy; data management & security; physical infrastructure; technical infrastructure; outreach and communications; operational environment; clinical/research infrastructure; novelty uptake & review. Descriptor summaries for each maturity model are given for each domain, as well as summaries of the overlapping areas. Not evaluated denotes that there were no associated descriptors found in that maturity model.

Federated Network	Governance	Collaborative Initiatives	Economics	Performance	Data Protection/Privacy	Data Management & Security	Physical Infrastructure	Technical Infrastructure	Outreach and Communications	Operational Environment	Clinical/Research Infrastructure	Novelty Uptake & Review
ELIXIR	Data Sharing Governance, Infrastructure & Initiatives Aligments	Data Sharing Agreements between nodes and Collaboration Agreements	Cost Plan, Secured Funding and Business Plan	Not Evaluated	Not Evaluated	Data Storage and security policies; authorization/authentication infrastructure for access control and permissions; data use ontology implementation; data discoverability policies and tagging; data reception mechanism; data standards; data quality control requirements; data quality control procedures	Secure data storage and API suppore; physical storage infrastructure; backup & data loss prevention; possible storage expansions	Data proccessing capacity, processing technical infrastructure and policies	Communication and outreach plans to encourage use of international infrastructure	Capacity to deal with ELSI issues, techical capacity, and training + capacity building for infrastructure within node and network	Technical infrastructure for data processing	Not Evaluated
FEGA	Dedicated Governance Bodies Strucuture within FEGA nodes and Strategies [Organizational]	Node Collaboration Agreements and Data Processing Agreements	Cost Plan, Short-term Funding and Sustainability Plan	Determined Key Performance Indicators	Data Protection Impact Analysis; Risk and Vulnerability Analysis	International best practiceses of data security; pre-emptive tagging of data for appropriate data use ontology; approval committee for data access; semi-automative application process for access; Risk register; SOP documentation for security breaches and incidents; data discoverability and tagging; data content quality assessment; data standards; metadataa harmonization; data flow	Can maintain or upgrade capacity when needed; periodic revision for staorage capacity planning based on KPI.	Adoption of common software and best practices; integration with EGA microservices/API ecosystem; compliance testing; stress testing; internal computing capacity; available network capacity; network reliability/security	Strategy, community outreach plans and adapted training material delivery to local users to use network	Training programmes for employees; internal SOP documentation and operation; external node interaction SOP; helpdesk SOP	Data standards and metadata management shold be implemented as workflows and automated as much as possible	Not Evaluated
B1MG	Dedicated Genomics Governances Strategies & Infrastructure in Healthcare [Organizational]	Data Sharing Policies and partnerships with research sector (industry and clinics)	Costed Implementation Plan (Strategy), Investments Plan, Access and Reimbursement Plans	Health Technology Assessment Framework for Genomic Tests, Cost-effectiveness of Genomic Tests, Societal Benefits consideration for economic modeling	Lawful Processing of personal data; Confidentiality of Patient’s Genetic Tests; Prevention of result mis-use; Consent; Norms and Regulations for use and sharing; Ethics	International best practiceses of data security; institutionalized data acesses strucuture; data structure	Not Evaluated	Not Evaluated	Awareness, strategies and community outreach plans to emphasize improtance of genomic integration into healthecare	Education, and career guidance/strategy for genomics in general curricula for health care professionals, providing flexible opportunities for continous training and career paths; Implementation of fully functional programs at a national level on genomic medicine	Data Processing and analysis infrastructure for genomics research, such as sequencing/genotyping, bioinformatics analysis, and etheir associated guidelines and structure	Uptake of novel tools for genomics, multi-disciplinary teams and ICT tool implementation for sake of genomics implementation into healthcare.
Common overlaps across all networks	Encourage international cooperation across nodes with agreements in place	Agreements to be made across nodes and stakeholders	Looks at cost of operations and ways to make operations sustainabile through funding or practices	No major overlaps but both FEGA and B1MG have periodic assessments to performance for future improvement	covers desensitization of relevant sensitive information that could be traced back to origin; should be in accordance to local/international privacy laws	covers data access rules and data management such as data/metadata structure and possible harmonization	Ensures data is physically safe, and can be uprgaded when capacity increase is required	Ensures data could be processed, especially for data management, as well as data security/protection could be maintained	Allows federated networks to share their objectives with the networks and ensures they have plans to be sustainable for their missions	Ensures the environment for the federated network could lead to the completion of goals and missions of the networks	Makes the stakeholders capable in using the data obtained from the networks in a interpretable way ensuring harmonization of standards for data and metadata	Most mature descriptor for majority of subdomains is to be open to evaluation and improvement for novel tools/practices

**Figure 4:**
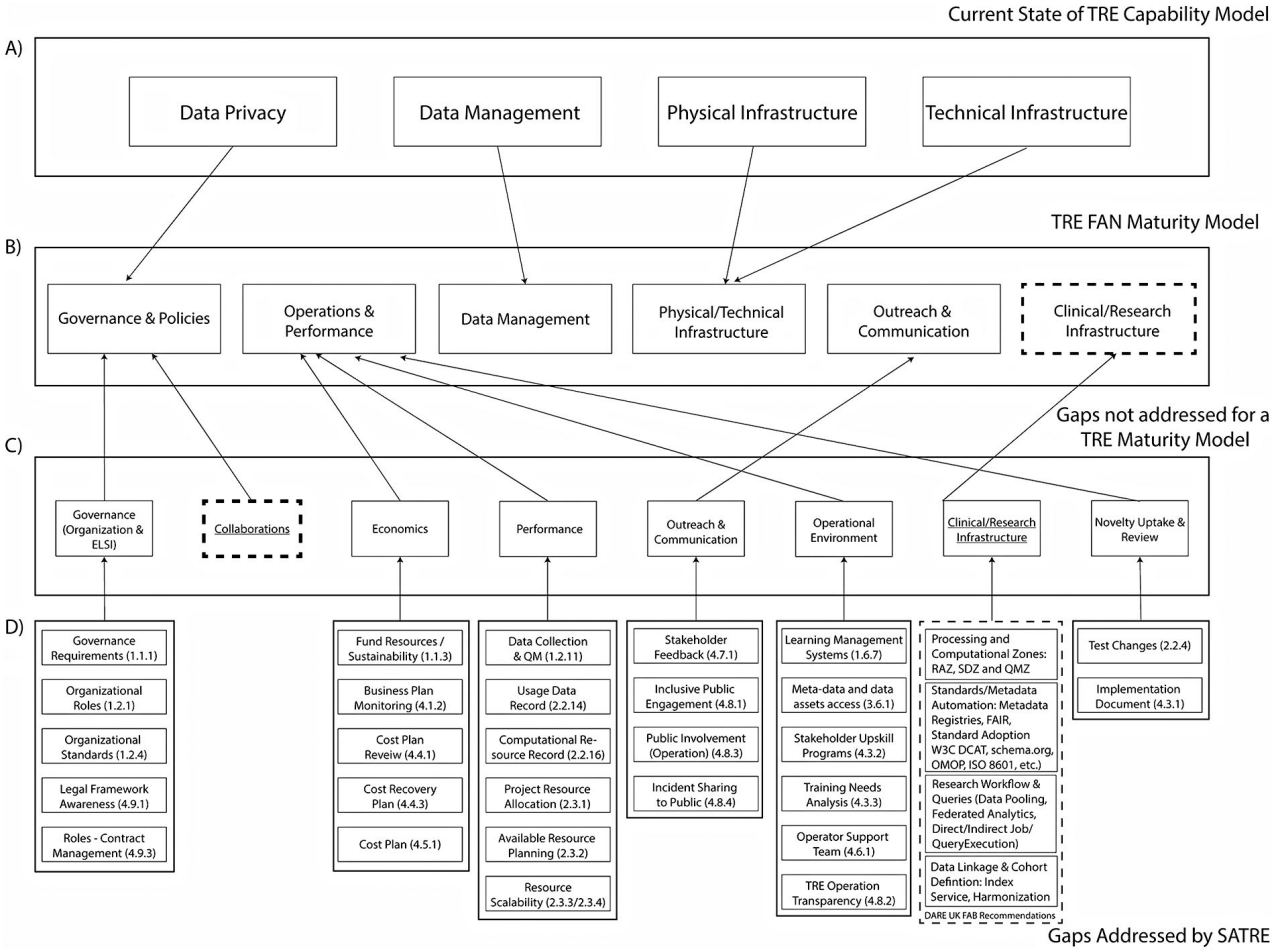
Development framework for a TRE federated network maturity model with Gap analysis using the capability maturity model. Figure illustrating the development of a proposed TRE Federated Network Maturity Model framework **(B)** derived from current TRE Capability Maturity Model descriptors **(A)** and incorporating identified gaps **(C)** and SATRE and FAB indicators **(D)** The model provides a comprehensive maturity assessment framework by incorporating existing components with gap analysis. The dashed line border indicates there are no suitable descriptors that were described by the TRE Capability Maturity model or SATRE, while arrows indicate overlap between the sections from the different documents.

As the Capability Maturity Model builds upon the defined fundamentals for TREs, it focuses on how to make the environment “safe” for data storage and access and does not include comparative analysis with other TREs as an evaluative tool. Figure 4 displays how HDR UK's Capability Maturity Model provides relevant attributes for TREs in the domains of data management, data privacy, physical infrastructure and technical infrastructure. However, TRE owners joining TREs networks would not only need to evaluate these domains, but also address the comparative gaps listed in Figure 4 to provide sustainability and continuity. Ultimately, these can be consolidated into the following six domains: Governance and policies, Operations and performance, Data management and security, Operational infrastructure, Outreach and communication, and Clinical/research infrastructure.

With these domains defining how best a TRE federated network might work, a maturity model of the six recommended evaluative categories can be recommended as a framework, upon which subsequent domains, subdomains, indicators and maturity descriptors could be added, refined and validated by the international TRE community (of which the HEALTHWISE Consortium is part of).

### Analysis of standard architecture for trusted research environments (SATRE)

The analysis of the SATRE TRE architecture spreadsheet checklist, which is a scoring evaluative assessment for individual TREs on information governance, computing technology and information security, data management, and supporting capabilities, has allowed for insights on the missing maturity model gaps that were indicated in Figure 4. The 160 implementation recommendations, with varying degrees of importance for a TRE, are rated as mandatory, recommended or optional. These recommendations have allowed for further elucidation on the operations of a TRE, and what also needs to be evaluated to be a successful network of connected TREs. General descriptors that could be allocated under the six domains were categorized accordingly as shown in Figure 4. Ultimately, the SATRE document provided a foundation to address the gaps that could not be fulfilled originally by the Capability Maturity Model, specifically in the domains of governance, economics, performance, outreach and communication, operational environment as well as novelty uptake and review. No suitable indicators were identified for the collaboration and clinical/research infrastructure domains.

### Analysis of DARE UK federated architecture blueprint (FAB)

The DARE UK FAB v2.2 contains 128 unique recommendations for enabling federation between TREs. These are classified on the level of importance for a TRE using the MoSCoW priorities of: must have, should have, could have, won't have. The recommendations are high-level and apply to the UK sensitive data federation ecosystem. Some recommendations are applicable to services beyond TREs, such as registries, and some refer to UK-specific accreditation requirements. While the aim of the FAB is to support connections between TREs, and the aim of the SATRE specification is to support establishing standalone TREs, there is significant overlap. 62 of the FAB recommendations map to at least one SATRE checklist indicators, with some FAB recommendations mapping to five SATRE checklist indicators (32). FAB recommendations were evaluated against the six domains to determine where they could fill gaps in the SATRE indicators using general non-UK-specific recommendations. Where there were partial overlaps with SATRE indicators, the more detailed and granular SATRE indicators were used. The FAB descriptors that could be allocated to the six domains are shown in Figure 4, with the FAB providing a foundation to measure maturity across the clinical/research infrastructure domain. Given the high-level FAB recommendations, we propose further clinical/research infrastructure domain indicators in the Discussion session, along with indicators for the remaining domain—collaborations.

## Discussion

### This study provides an initial maturity model for federated networks of TREs

Maturity models provide comprehensive frameworks for organizational capabilities through the assessment of multiple organizational dimensions, by ranking specific items by levels of maturity. Assessment and benchmarking establish baseline capabilities and enable comparisons between institutions, directly providing feedback that can guide resource allocation and improvement initiatives. This supports a systematic improvement process, while providing structured pathways for advancement. The organizational analysis also encourages further development through cultural transformation, skills building, and knowledge transfer, while maintaining quality assurance through established standards and best practices. The assessment of these interconnected components creates a holistic approach to measuring, planning, and achieving organizational maturity, ensuring consistent performance and continuous improvement across all operational aspects.

Based on the results outlined in Figure 4, where we identified areas of overlaps and gaps across existing maturity models, the following sections consolidate the comparative observations across the six proposed domains of the TRE federated network maturity model.

### Governance and policies

The consolidated domain of governance and policies should include the principles for the original governance, collaborative initiatives, and data privacy aspects. The evaluation of this domain should provide a structure for TREs networks to assess their operational structure, personnel roles, and have preliminary approaches to ELSI issues. However, as HDR UK's capability maturity model looks mainly at how to provide the SAFE principles and not how to operate a TRE from top to bottom, the concepts and details for governance and policies were not considered for the model.

The evaluation of governance should allow for the TRE to compare its maturity as an organization, making sure it can operate as an entity within its space of interest. Having governance requirements, organizational roles and standards elevates the organization's own awareness regarding its operation (Table 2). Aside from the individual TRE nodes, a TRE network might also have to acknowledge the capacities of the individual TREs within the context of the network, for example, whether it will be able to provide not only data, but also the resources needed to have analytics capabilities.

Individual TREs within such a network can also become more sustainable by reviewing their policies and agreements, possibly providing greater sustainability through a lowered costs base. Policies and workflows should encourage collaboration on projects and activities, aligning with the mission/objectives of individual TREs. Aside from TRE inter-node activities, TREs should also evaluate how they can best support local or international academic and industrial partners, providing further collaborative growth opportunities. Adapting existing frameworks and output standards can also prepare entities for cooperation, such examples could be observed through ELIXIR's maturity model to evaluate its own entities for their alignment to 1 + MG's roadmap and trust framework, both the European Health Data Space (EHDS) and European Open Science Cloud (EOSC) outputs, and the National Genomic Programme's activities. These alignments will allow for easier harmonization within their activities, as well as continued advancements through future collaborations. As all three of the federated networks placed emphasis on achieving their mission and objectives in data sharing, the most mature level in the maturity models specifies the phrase to “support international cooperation”.

Data privacy and compliance with data security laws of the country and/or regions are especially important, ensuring proper handling of data, as well as requiring ongoing processes to assess and review adherence to relevant local and international data privacy laws. Laws such as the General Data Protection Regulation (GDPR) act of the European Union, Health Insurance Portability and Accountability Act (HIPAA) of the United States, and China's Personal Information Protection Law and Data Security Law will limit what data can be stored, and demand consent related requirements, cross-border restrictions and data processing regulations. ELIXIR is a consortium of nodes from different nations and the European Molecular Biology Laboratory (EMBL). EMBL allows ELIXIR to use its legal personality as an international organisation. Although EMBL and ELIXIR are not subject to GDPR, they are governed by Internal Policy 68 which enables ELIXIR to align its practices with those of its European members (46). Lastly, an important concept that is introduced from HDR UK's capability maturity model is automated statistical disclosure control within the most mature tier. Sensitive data should be unidentifiable and thus it should not be possible to link it to an individual (47). When measures to help ensure TREs fulfill their legal obligations are automated, or semi-automated, the human error risk is reduced from an otherwise manual process.

ELSI considerations will also require members of the public to be aware of the operations and be capable of taking organizational roles within the structure of the institutions. Under SATRE, members of the public should be included in TRE operations and/or have roles that oversight operations, either through steering groups or project approval panels (42). In extension, ethics review boards should also be considered for the governance of such TREs and networks for ensuring research and operation ethics are the norm, as evaluated by the B1MG network. Similarly, the ethical use of AI in healthcare under the EU AI Act, as well as in the EHDS, requires additional scrutiny, acknowledging ethical principles such as explainability, transparency, trust, privacy and safety to strengthen the understanding of how AI use is governed and utilized for the interest of engaged stakeholders while also safeguarding against the potential harms of AI use in healthcare (48). The correct integration of such technology can affect how accepted its use is within collaborative networks, and conversely, could be limiting if left unused. The EHDS will provide European citizens empowerment with what data is shared with healthcare providers and physicians, as such compliance in terms of policies will have to be prioritized so that potential stakeholders will be confident in the correct usage of healthcare data by such a network. Services and operational activities of TREs locally and internationally can be impacted by such laws.

### Operations and performance

The consolidated domain of Operations and Performance should consist of evaluative measures to observe the maturity of the TRE's operation and practices, especially to determine its sustainability, as well as fluent setup and management of resources. The subdomains include economics, performance, operational environment and novelty uptake/review, which are factors that contribute towards a successful entity for its business and function.

Economics of TREs can be impacted by various factors and would require assessments on how mature the economic planning and execution of individual TREs are. The sustainability of TREs will largely be entity dependent. TREs can be government supported, or privately owned entities. However, each entity should evaluate how it operates to be capable of supporting itself as an entity. Funders can be sector dependent, and TRE entities can be tailored for different sectors, from academic, charity, public, non-governmental organizations (NGO), and consortiums (49). Depending on the TRE's function, e.g., if it only performs data storage or has external practices that aid in the TRE's operations, the expenses and income of the TRE can differ as well. Different TREs can have very different costs, investments and funding plans, so how to best make the entity sustainable to support research must be evaluated. Standard expenses such as equipment and infrastructure, supplies, software, hardware, human capital and resources, facilities upkeep and maintenance, operational and compliance, can all impact the burden of operation. These expenses can also vary between locations, regions, and countries based on local standard of living, investments, currency and partners. The amount of funding received can also differ between TREs. As such, finding the best suited method is a priority. Business plans, such as initiating with potential stakeholders to provide services, gaining license fees if research uses associated data, patent ownerships, subscription fees, sale of data usage, can all become possible ways for TRE’s to gain income to be used to invest back into itself for operation. Outside investments could also be made to subsidize its own operation. How specialized or how useful the data collection of a TRE is can impact how much investment a TRE can bring in, with factors such as societal pressures, cohort importance, and trends in non-communicable and transmissible diseases all deemed important considerations.

Performance is evaluated by tracking specific key performance indicators (KPIs). Examples of KPIs include users per year, concurrent users, usage time, total queries, query volume, bandwidth used, resource utilization, response time, database growth rate, and others. These indicators are also relevant in the case of providing statistics to gain external funding, investments, business and collaborative agreements. KPIs on academic outputs (e.g., publications, posters, patents, tech transfer agreements and acknowledgements) allow TREs to evaluate their success within the research landscape. In terms of operational environment KPIs, the example of B1MG is its emphasis on sponsoring courses and educational training in genomics, while also advocating for genomics integration in general educational curricula, and the career progression of their trainees. FEGA through their maturity model also evaluate the interactions between nodes, centralized nodes, and external entities, having SOPs for different types of interactions, ensuring that activities are standardized as much as possible to establish workflow and measurable outcomes. This also includes helpdesk staff and staff training on how best to communicate with their partners and stakeholders.

Evaluating the novelty uptake, (i.e., creation and/or integration plans of new analytical tools and software) can allow the TRE to provide an added-value indicator. Although only B1MG had evaluated this subdomain directly, ELIXIR and FEGA all have descriptor words within their maturity model to review the landscape regularly, and implement new tools and developments, especially to initiate international collaboration activities.

### Data management and security

A major component that is covered by the HDR UK capability maturity model and the DARE UK FAB, is the management of TRE data ingress and egress, internal and external data management processes, data access procedures, and data security. A TRE's commitment to provide data security, privacy and data use monitoring will be what draws its potential stakeholders to utilize their services, and outlining how it does so is important to provide trustable data (50). There can always be debates on the perceived levels of trust, but outlining how the environment is secure provides crucial information for individuals to decide if the platform is valuable to their needs, as well provide the public evidence that the data can be stored there with trust (51, 52). Discussion of whether AI could be effectively used to perform data management will also require further conclusions, as stringent privacy regulations can restrict how sensitive data is used in learning systems or processed at scale. As such, whether AI could streamline data de-identification, annotation and interpretation is uncertain, where these processes will still require rigorous quality control. Ultimately, the challenges make it unclear whether complex AI implementation will truly be superior compared to automated tools that produce standardized outputs. Beyond security risks, primary hurdles in AI adoption are still the potential hallucinations and erroneous interpretations, which could lead the technology to be counterproductive without robust validation of its outputs and outcomes.

Various foundational features were integrated within the different maturity models, such as methods to ensure data security, restricting data access, acknowledging data flow protocols, data processing requirements and standards for data structure. Determining the maturity of a network's data security includes evaluating the network and security controls by both hardware and software means, as well as security breach prevention, and emergency response protocols. Implementation of user control such as federated single sign on, or user passports and visas through institutional access with appropriate access applications confirmed by data access committee can all contribute to ensuring data safety (40). Other related security measures include remotely accessed software defined provisioned workspaces, browser-based access, and export restrictions all limiting unauthorized data usage (40).

### Operational infrastructure

In the operational sphere there is an overlap of the maturity and capacity model: the maturity model assesses an organization's ability to manage and utilize health data effectively, while the capacity model looks at the human and technical capacity required to support data-intensive research. Both lenses were taken into consideration in this analysis, as they address different, complementary aspects of data-driven research.

Thus, the operational infrastructure covers both physical and technical infrastructure, although not evaluated by all maturity models of interest. This covers the physical hardware components [e.g., hard drives for storage and backup, computer processing units (CPUs), graphics processing units (GPUs), networking equipment for cloud services], and the physical computers for direct access/remote access. Its maturity ensures that data is physically safe and has mechanisms to prevent data loss.

The technical infrastructure involves the components needed for operation and management of the information technology and operational systems. Application programming interfaces and microservices for example, might be utilized to track data usage and access, including comprehensive request logging, authentication monitoring, and usage metrics to ensure system reliability, security compliance and data flow managing.

The FEGA maturity model requires an operational conditions review, such as compliance testing of different agreements and implementation by different ecosystems, stress tests of equipment and network, computing and processing capacity, and network security. Security information and event management, and risk management review protocols should also be in place to minimize potential security breaches or failure events in the case of a TRE network.

### Outreach and communication

Outreach and communication enable the local and regional community to understand the value of the TREs mission and objectives, encouraging local and regional partnerships and collaborations. This could be observed through the B1MG's maturity model, engaging in strategies that can reach their goals in genomics awareness and acceptance. Similarly, ELIXIR aims for its users to actively use services from the international infrastructure, while FEGA encourages internode data sharing activities. A mature TRE network that can involve public/stakeholders' awareness of services and data stored, as well as their relevance, greatly increases its potential operational activities. Transparency on organizational activities, reports on project outcomes, and establishment of feedback channels can enable stakeholders to keep informed about the work and support understanding of their needs and concerns. Having ways to ensure the stakeholders are engaged, and ways to keep the TRE accountable for the data it holds, can also build trust between society and the scientific community, furthering the understanding between all parties when utilizing clinical data as well as the expectations when using such data (51).

Providing guarantees that related clinical data is protected through informed consent, as well as ensuring that the data cannot be used for discrimination or stigmatization via employment or insurance, can encourage patient engagement and can increase in related data consent, and thus increased data collected (52). However, consented data in clinical research can be subject to biases, since patients who agree to participate in research are not always representative of the general population, where those with specific diseases are more likely to provide consent than the general population (53). Unconsented population-level analysis on de-identified data can work for projects in the public interest, can bring significant societal benefit, and minimize biases, as long as participants are informed about usage, and research status is transparent (54, 55). A key driver for the use of TREs in the UK is to enable projects using unconsented data.

The importance of stakeholder engagement is exemplified by successful pilot implementations like Portugal's Administrative Modernization Agency's Mobile Key Solution public service chatbot to access public services. Potential inaccuracies are explained and understood by users who opt to use this service rather than use the phone or queue up at public sector buildings. This demonstrates how transparent stakeholder involvement can facilitate acceptance of innovative data-driven solutions while delivering tangible societal benefits (10).

### Clinical/research infrastructure

This last domain is the hardest to evaluate per TRE, as different TREs can have different objectives. As such, it should provide the foundation upon which TRE specifics are built on that make it relevant for TRE implementations in either clinical or research settings. Example implementations include the HDR UK cohort discovery service. Without directly providing the user with raw data, metadata is browsable through an interface that searches a network of data providers to enable users to discover datasets of interest. Interoperability across the network is achieved through a consistent use of data standards.

Emphasis on FAIR (Findable, Accessible, Interoperable, Reusable) data principles requires workflows and protocols in place to make sure data made accessible for research has standardized formats and associated metadata. This will support consistent and reproducible analytics across federated TREs (35, 56). The workflows, protocols, best practices and standards also aid in the transparency of research, providing reproducible data that could be validated. Publicized open standards and workflows enable organizations to understand the formats, ensuring the data being accessed fit quality control requirements. The automation, or semi-automation, of statistical disclosure control prevents release of potentially sensitive outputs, while reducing the burden on TRE owners to check outputs manually (27, 47, 57).

Proper tagging of metadata enables data discovery tools and dashboards to be utilized for data browsing, such that new and suitable data sources can be found for big data projects. Metadata catalogs and cohort discovery tools, like those provided by HDR UK, eases discovery and access of specified data enabling researchers to find, and request access to, the associated raw data (58). The DARE UK FAB recommendations define the use of identifiers that are globally recognizable within the federation at the project and dataset levels. The UK Health Data Research Alliance recommendations for a data use register standard supports organizations with providing a public record of how data is being used for research, by who and for what purpose (59). Overlapping with the objectives of the outreach & communication domain, it is anticipated that the use of such a data use registry will improve transparency and build public confidence around the use of sensitive data for research. Finally, the Data Use Ontology (DUO) tagging of datasets has been included by FEGA to authenticate usage conditions for access control. Providing relevant but cost-effective processing capabilities would also be important based on the requirements and resources of the users, as the service provided must have value to promote its usage (9).

### Considerations for a federated network of TREs

The SATRE and FAB recommendations, although not in the format of a maturity model, contains descriptors and an optionality status for each item, providing good technical indicators of how to operate a TRE standalone and in a federated network. These indicators can be used as a foundation for a potential TRE network maturity model. However, while SATRE provides guidance for each detailed recommendation, the FAB recommendations are higher-level, with up to five SATRE recommendations mapped to one FAB recommendation. Reviewing and assessing the maturity of federated networks will require the maturity model to provide guidance for relevant FAB recommendations, where there is no overlap with SATRE, to detail the options available for implementation.

One area to be defined is how TREs will effectively support each other while maintaining their individual operations. The “Collaboration” domain was identified as a gap not addressed by the TRE Capability Maturity Model, SATRE or FAB (Figure 4). The feasibility of running multiple TREs under a single infrastructure or software stack requires careful consideration for standardization and efficiency. Furthermore, multidisciplinary experts and teams will be required to integrate the pre-existing data, as well as understanding what tools and analysis scripts to be provided, as shown by Kroes et al.'s efforts in harmonizing unstandardized disease registries to be used for federated data analysis (60).

The protocols and mechanisms that can be established to initiate and sustain collaborative activities between TREs should be defined. Incentives might be needed for the recruitment of new participating nodes within a network, with further incentives required to initiate collaborations between the TREs within such a network (34). However, incentives such as funding or academic rewards will require external structure or internal agreements that could impact a network's sustainability without engagement from potential stakeholders. The prestige from networking and publishing might not be enough, as potential members have external financial pressures and efforts spent deciding how best to incentivize such collaborations can affect the overall success of such networks. Lastly, will a facilitating/centralized node be necessary, and if so, what are the essential requirements for a centralized node that could coordinate and facilitate these inter-TRE relationships while ensuring consistent implementation of the maturity model to ensure quality data and analytics capacity. Resolving the responses to such questions will dictate the feasibility of establishing and growing such a network, as well as the willingness of TRE participation into such an initiative. Current work by the HDR UK Health Data Research Gateway, European Network of Trusted Research Environments, and the Australia Research Data Commons will largely be referenced in the future to initiate best federated network standards and practices for TREs (22, 61–63). Furthermore, work performed by Lannon et al. on maturity grid assessment tools for learning healthcare networks are also largely applicable (64). They describe self-assessment systems that allow users to analyze their place within collaborating networks, with case studies on how specific users use their assessment tool to score themselves on particular domains, including technical framework requirements in security and interoperability.

Clinical/research infrastructure as a domain will also need to be further elucidated but is further dependent on the objectives of such a network of TREs and its desired role in supporting sensitive data research. For example, B1MG has set up their maturity model to aid advancements in personalized medicine through genomics research and has required maturity evaluation of its use of standards in genomics to aid genomic research initiatives and projects. The evaluative domains should provide progress towards the long-term goals of the network, and acknowledging the objectives and missions of such a network will help with building the foundation and advancements for the clinical and research landscape allowing the network to position itself as being an important resource.

## Challenges

There will be challenges for low and middle-income countries (LMICs) associated with joining international federated networks of TREs. Finding comprehensive solutions to cost effective technology, sustainable funding models and local capacity building will be required to initiate continuous partnerships with LMICs. For example, LMICs which have unreliable internet connectivity and/or computational resources will struggle to ensure a full suite of data protection requirements as a TRE network may not allow for possible non-secure connections due to increasing security vulnerabilities (65). Among 80% of research on global diseases originates from high-income settings, and this can theoretically be increased, based on collaborative activities as part of a TRE network (58). Thus, the challenge can also be described as the ecosystem or state of healthcare research data in different nations, affecting the readiness for data to be reused. Also, from an outreach and communication perspective, involving LMICs in the work can have a great local impact as policymakers may take note of the findings or be inspired to engage in ongoing research (66). This impact though needs to be demonstrated clearly and unequivocally.

The economization of health data as a strategic resource also presents itself as a challenge, as nations see health data as valuable assets that can provide economic benefits and strategic advantages (67). This perspective has led to a stringent regulatory framework and legal barriers that prohibit cross-border data transfers without government involvement. TRE networks need to acknowledge these challenges and incorporate them into their operational design. Also, different countries bearing different privacy laws and data policies could impact the everyday operation of TREs within such an international federated network, as well as having organizational roles that keep up with the legality of its everyday operations/inter-network activities.

Lastly, the resources it takes to operate a large-scale federated network of TREs that serves as a global data access platform will be significant, especially concerning operations and funds. The scale of such networks will require further elucidation of their feasibility, as the larger scale operations such as enabling data to be used for federated learning will require that all data that is used for model training to be valid in terms of standardization and quality (27). Since different TREs are likely to have varying levels of maturity, integrating them seamlessly into larger federated networks presents significant operational challenges. The activities and timescales involved in scaling such a network - from a single node to a full federated analysis - will need careful consideration in terms of purpose and feasibility. This will require further investigation into the technological and infrastructure capabilities of existing TREs and federated networks in order to draw meaningful conclusions. A multi-level maturity model will provide a pathway to technical maturity that can be followed by prospective partners in a TRE federated network.

## Conclusion

As healthcare research demands have increased, driven by technological advancements, TREs have emerged as solutions to share sensitive clinical and biological data while safeguarding privacy and data integrity. Some of the key TREs principles include enabling controlled access, maintaining data sovereignty, and implementing protocols for interoperability across various data sources. The maturity of such TREs can be evaluated using maturity model domains from entities like ELIXIR, FEGA, and B1MG. However, as TREs are forming international federated networks, there exists no maturity model for a TRE network.

We have compared existing federated entity maturity models, identified potential gaps and have recommended a novel maturity model framework, applicable to a federated network of TREs. This new maturity model framework, as presented in Figure 4, focuses on optimizing robust governance, technical infrastructure, while addressing legal, ethical, and operational challenges.

## Data Availability

The original contributions presented in the study are included in the article/Supplementary Material, further inquiries can be directed to the corresponding author.

## Members of the HEALTHWISE Consortium

Tim Beck, University of Nottingham, UK; Jillian Beggs, University of Dundee, UK; Angela Chee, Institute for Infocomm Research, A*STAR, Singapore; Samson Chow Ching Po, National University Hospital, Singapore; Jason Ferris, University of Queensland, Australia; Mallory Freeberg, EMBL-EBI, UK; Kate Frost, Nottingham University Hospitals NHS Trust, UK; Koh Furuta, Chiba Medical Center, Japan; Carole Goble, University of Manchester, UK; Alain-Dominique Gorse, University of Queensland, Australia; Emily Jefferson, HDR UK, UK; Neerja Karnani, Bioinformatics Institute, A*STAR, Singapore; Thomas Keane, EMBL-EBI, UK; Zisis Kozlakidis, World Health Organization, France; Birendra Kumar Yadav, Biobank India Foundation, India; Valentina Martufi, Center for Data and Knowledge Integration for Health (Cidacs/Fiocruz Bahia), Brazil; Sebastian Maurer-Stroh, Bioinformatics Institute, A*STAR, Singapore; Khin Mi Mi Aung, Bioinformatics Institute, A*STAR, Singapore; Nicola Mulder, University of Cape Town, South Africa; Soichi Ogishima, Tohoku University, Japan; Chris Orton, Swansea University, UK; Sumir Panji, University of Cape Town, South Africa; Helen Parkinson, EMBL-EBI, UK; Neil Postlethwaite, HDR UK, UK; Marie Quinlan, Nottingham University Hospitals NHS Trust, UK; Philip R Quinlan, University of Nottingham, UK; Stian Soiland-Reyes, University of Manchester, UK; Clair Sullivan, University of Queensland, Australia; Simon Thompson, Swansea University, UK; Dennis Wang, Bioinformatics Institute, A*STAR, Singapore; Kiraely Adam Wong Zhongren, Singapore Translational Cancer Consortium, Singapore; Anne Wozencraft, HDR UK, UK; Xing Yi Woo, Bioinformatics Institute, A*STAR, Singapore; Yi-Hsin Yang, National Institute of Cancer Research, Taiwan; Guido Zuccon, Queensland Digital Health Centre, Australia.
