# Supplementary material for: A maturity model framework for federated networks of trusted research environments
Source: Front Digit Health. 2026 May 15;8:1699125. doi: 10.3389/fdgth.2026.1699125 (PMC13219306; doi:10.3389/fdgth.2026.1699125)
Supplement: Supplementary file 1 [file Table1.docx]

| **Maturity Level** | **B1MG Maturity Level Model** | **ELIXIR Human Data Infrastructure Maturation Model** | **Federated European Genome-phenome Archive (FEGA) Maturity Level Model** |
| --- | --- | --- | --- |
| Level 1 | Ad hoc | Requirements being gathered | No specific actions taken towards a fully operational node |
| Level 2 | Defined at local level | A plan is drafted to implement | Being aware of the needs and initial actions taken to become a node of the federation |
| Level 3 | Documented, functional, monitored | … is deployed | Working to be minimally operational |
| Level 4 | Adopted by national/regional health systems | … is enforced and reviewed regularly | Entering full operational mode |
| Level 5 | Adoptable to opportunity and change, supports international cooperation | N/A | Keep your full operational mode with periodic reviews of the status |

**Supplementary Table 1.** General descriptors from the B1MG, ELIXIR and FEGA Maturity Models.
